# Coupling of machine learning and remote sensing for soil salinity mapping in coastal area of Bangladesh

**DOI:** 10.1038/s41598-023-44132-4

**Published:** 2023-10-10

**Authors:** Showmitra Kumar Sarkar, Rhyme Rubayet Rudra, Abid Reza Sohan, Palash Chandra Das, Khondaker Mohammed Mohiuddin Ekram, Swapan Talukdar, Atiqur Rahman, Edris Alam, Md Kamrul Islam, Abu Reza Md. Towfiqul Islam

**Affiliations:** 1 Department of Urban and Regional Planning, Khulna University of Engineering & Technology (KUET), Khulna, 9203 Bangladesh; 2 Department of Geography, Texas A&M University, College Station, USA; 3 Population Health Sciences, Harvard University, Cambridge, USA; 4 Department of Geography, Faculty of Natural Sciences, Jamia Millia Islamia, New Delhi, 110025 India; 5 Faculty of Resilience, Rabdan Academy, 22401 Abu Dhabi, United Arab Emirates; 6 Department of Geography and Environmental Studies, University of Chittagong, Chittagong, 4331 Bangladesh; 7 Department of Civil and Environmental Engineering, College of Engineering, King Faisal University, 31982 AlAhsa, Saudi Arabia; 8 Department of Disaster Management, Begum Rokeya University, Rangpur, 5400 Bangladesh; 9 Department of Development Studies, Daffodil International University, Dhaka, 1216 Bangladesh

**Keywords:** Environmental sciences, Natural hazards

## Abstract

Soil salinity is a pressing issue for sustainable food security in coastal regions. However, the coupling of machine learning and remote sensing was seldom employed for soil salinity mapping in the coastal areas of Bangladesh. The research aims to estimate the soil salinity level in a southwestern coastal region of Bangladesh. Using the Landsat OLI images, 13 soil salinity indicators were calculated, and 241 samples of soil salinity data were collected from a secondary source. This study applied three distinct machine learning models (namely, random forest, bagging with random forest, and artificial neural network) to estimate soil salinity. The best model was subsequently used to categorize soil salinity zones into five distinct groups. According to the findings, the artificial neural network model has the highest area under the curve (0.921), indicating that it has the most potential to predict and detect soil salinity zones. The high soil salinity zone covers an area of 977.94 km^2^ or roughly 413.51% of the total study area. According to additional data, a moderate soil salinity zone (686.92 km^2^) covers 30.56% of Satkhira, while a low soil salinity zone (582.73 km^2^) covers 25.93% of the area. Since increased soil salinity adversely affects human health, agricultural production, etc., the study's findings will be an effective tool for policymakers in integrated coastal zone management in the southwestern coastal area of Bangladesh.

## Introduction

The deterioration and desertification of land due to salinization is a long-standing issue in the environmental history of the planet^1^. Because of many hydrological processes, salinity, or a high concentration of mineral salts in water or soil, may be defined in many ways^2^. One may classify it as either main or secondary^3^. Both natural events (such as floods and storm surges) and human activities (such as excessive fertilizer usage and poor agricultural land management) may cause salinization, although the latter is more common^4^. Soil salinity has far-reaching effects all throughout the world. The United Nations' Food and Agriculture Organization (FAO) estimates that salinity affects about 424 million hectares of top soil (0–30 cm) and over 833 million hectares of subsoil (30–100 cm), or about 3% and 6% of the world's topsoil and subsoil, respectively^5^. A worldwide increase of 2 million hectares each year, salinization has already impacted over a hundred nations^6, 7^. Due of soil salinity’s negative effects on agricultural output, it poses a danger to both world food security and agricultural earnings. There is an annual loss of US$12–27.3 billion owing to low agricultural output throughout the world^8^. Nevertheless, the extent to which salt hinders agricultural output varies greatly depending on various parameters, such as soil texture and water content, soil nutrient status, plant species and variety, development stage, insect pressures, and the ions contributing to salinity^9–12^.

Various studies have been done all over the world regarding ecology, environment and disasters for example: modelling the morphometric characteristics and course of Brahmaputra river^13^, modified managed aquifer recharge model for groundwater management^14^, geospatial approach for developing an integrated water resource^15^, unique approaches to water management^16^, development of stormwater pretreatment system^17^, opportunities and challenges for implementing managed aquifer recharge models^18^, hydrology, water quality and trophic state of Pergau Reservoir^19^, prediction of drought using machine learning algorithm, estimating solid waste generation and suitability analysis^20^ etc. Numerous studies have been done regarding salinity which is an important topic relevant to ecology, environment and geospatial science. GIS and remote sensing, statistical analysis and machine learning algorithms etc are the commonly used method to detect salinity. Least square regression^21^^,^^22^, partial least square regression^23^, developed linear regression model^24^, pearson correlation coefficient analysis^24^ etc. are the most frequently statistical method. GIS and remote sensing has become very popular in terms detecting soil salinity over the last couple of years^13, 25–28^. Soil salinization monitoring and mapping are only two of the many uses for Sentinel-2^29, 30^. It has a high spatial resolution, several wavebands, and a relatively quick return time. The Multi Spectral Instrument (MSI) outperforms the Operational Land Imager (OLI) in terms of soil salinity tracking due to its high spatial and temporal resolution, but the OLI overestimates the area of salinized land and the area of salinized land covered by vegetation^21^. Compared the accuracy of the two sensors in estimating farmland soil salinity and found that these two sensors achieve similar Soil salinity mapping was performed using the OLI and MSI^31^, and the results demonstrated that various salinity levels in different electrical conductivity (EC) ranges may be deduced using regression analysis of ground-measured and satellite data. The identification of soil salinity using GIS and remote sensing data may be constrained by factors such as spectral variability, vegetation cover, air interference, and the availability of ground-truth data^32^. These constraints can be mitigated through the utilization of many sensors, incorporation of vegetation indices, implementation of atmospheric correction algorithms, and augmentation of ground-truth data collection^32^. Furthermore, the utilization of machine learning techniques, the analysis of time series data, and the incorporation of hyperspectral data have the potential to enhance the precision and reliability of soil salinity detection^33^.

There has been a growing interest in using machine learning models for more accuracy in term of salinity mapping. Several machine learning models such random forest^33^, support vector model^34^, decision tree model^35^, artificial neural network etc have been used to detect and for mapping soil salinity in different regions of the world. The performance of different machine learning algorithms for soil salinity mapping can vary depending on a number of factors, including the data characteristics, the model complexity, and the specific algorithm used. The performance of a machine learning algorithm might be influenced by the particular algorithm employed. Various algorithms are more appropriate for specific categories of issues. For instance, Artificial Neural Networks (ANNs) are commonly seen as more suitable for addressing problems characterized by intricate nonlinear linkages, whereas decision trees are frequently deemed more appropriate for problems with simpler relationships. ANNs are often considered to be more accurate than other machine learning algorithms for soil salinity mapping because they can learn complex nonlinear relationships between soil salinity and other environmental factors^36^. However, ANNs can also be more complex and time-consuming to train than other machine learning algorithms^37^.

Salinity intrusion has been increasing in frequency in recent years in Bangladesh. The climate around the Bangladeshi coast is notoriously cyclical. Despite the fact that just around 20% of the nation is located along the coast, of which roughly 53% is impacted by varying degrees of salt^38^, water and soil salinity have grown at an alarming pace over the previous few of decades. Increase in soil salinity in Bangladesh is being caused both by the effect of the changing climate, geographic location, deforestation, as well as human activities, like extreme level extraction of ground water, unplanned agricultural pursuits (shrimp farming, etc)^39^. Two major aspects of salinity intrusion are particularly important for the coastal people of Bangladesh: (a) the productivity of saline-affected people’s food security and livelihood, and (b) the salinity intrusion causing significant problems with safe drinking water, which affects people's health, especially women’s health. Besides, rising salinity also has adverse effects on the agricultural sector, forestry, the industrial sector, and the supply of safe drinking water^40^. There was a 21% rise in agricultural output loss due to rising salinity in 1976, as reported by^40^, which equates to an estimated loss of 647,000 tonnes. Salinity intrusion reduces the production of vital crops like rice and vegetables, coconuts, betel nuts, fruit, and even grass and hay for cattle, all of which are often grown in the coastal zone. It is also responsible for the extinction of native fish populations, and the contamination also spreads to both surface and groundwater, making it unsafe for human consumption^41^. Inadequate access to clean water threatens public health and safety, especially for women and girls. Additionally, the lives of people and animals in the area of the Sundarbans, the world’s largest mangrove forest, as they are gravely threatened by the incursion of salt water into inland freshwater sources. Satkhira the south west region of Bangladesh is facing a huge problem regarding salinity. Satkhira is facing soil salinity due to its geographic location, low-lying topography, overuse of groundwater, converting crop fields to shrimp farming and deforestation. Individuals across different age groups are currently encountering a diverse range of health-related issues due to the presence of salinity in the region of Satkhira. In addition, soil salinity has had a substantial impact on agricultural production. A drop in yields of traditionally farmed items, such as rice and vegetables, has been observed in the region mostly as a result of soil salinity, which poses a significant risk to these crops due to their susceptibility to salt damage. Consequently, local agricultural practitioners have been pushed to transition towards cultivating salt-tolerant crops such as prawns and crab. The research conducted by^42^ in Satkhira has revealed that salinity has adversely impacted 62.3% of the local population's conventional and historical methods of sustenance, leading to the emergence of alternative livelihood strategies such as day labor, crab fattening, and shrimp farming. Nevertheless, because to the significant sodium content, these other options are also encountering difficulties. The procedure for mapping soil salinity holds significant importance in the vulnerable coastal area of Satkhira^26^. By discerning areas characterized by elevated salt content, farmers are empowered to make informed choices regarding the appropriate selection of crops and irrigation methods. The aforementioned procedure aims to mitigate agricultural losses, streamline land-use planning, improve soil and water management techniques, and aid in adapting to the consequences of climate change. Mapping plays a crucial role in safeguarding agricultural productivity and environmental sustainability, as well as enabling efficient resource allocation for sustainable development in light of the challenges posed by escalating sea levels and changing climate patterns.

Many researchers have performed different studies related to soil salinity in Satkhira region.^27^ have used partial least regression for soil salinity,^26^have used satellite images for the detection of salinity,^42^ have detected the salinity related problems,^26^ have done salinity based land use zoning, soil salinity and its relation to other properties has been analyzed by^43,44^ have analyzed the salinity level in Khulna, climate change induced salinity intrusion by^45^, some studies have shown the impact of salinity in livelihood^46^ etc. However, these studies have yet to use machine learning algorithms to detect and mapping of soil salinity in the Satkhira district. This research aims to fill this gap. This study will use three machine learning algorithms, namely random forest (RF), bagging with RF, and artificial neural network (ANN), to detect and map soil salinity using thirteen different indicators and ground samples. Specific objectives of the study are: (i) detection and mapping of soil salinity using three different machine learning algorithms, (ii) validation and accuracy assessment of three different machine learning models, (iii) selection of the best-performed model based on relationships with indicators and accuracy assessment. The present study has the potential to make a valuable contribution to the global body of knowledge by addressing a significant research gap in the mapping of soil salinity in the Satkhira district. This research endeavors to enhance the precision and effectiveness of soil salinity mapping in the region through machine learning methods. Implementing these measures can mitigate the likelihood of crop impairment, improve water resource utilization efficiency, and safeguard the ecological diversity within the Satkhira district. The research findings can serve as an increased knowledge regarding soil salinity and its consequences in the Satkhira district. This may facilitate the mobilization of farmers, policymakers, and environmental managers in the area to undertake measures to mitigate soil salinity.

## Methods and materials

### Description of study area

**Figure 1:**
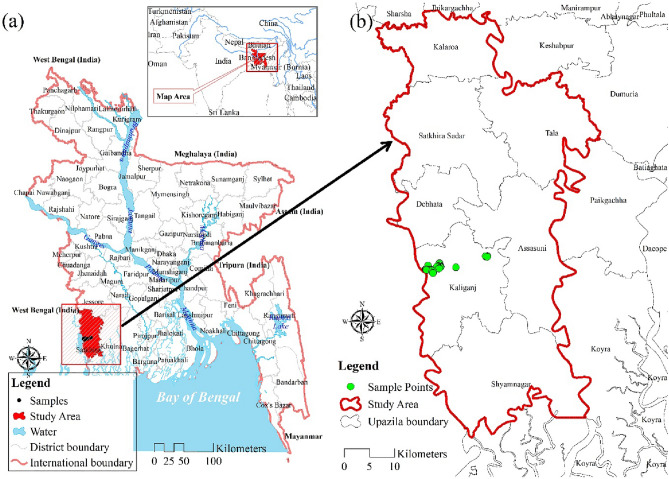
(**a**) Location and (**b**) Upazilas of the study; prepared by the authors using ArcGIS software version 10.5, (https://www.esri.com/en-us/arcgis/products).

The southern coastal regions of Bangladesh, which consist of 19 districts, are mainly vulnerable to soil salinity. Among all the districts of the southern region, the condition of Satkhira district is the worst. Every year, this district is severely affected by natural calamities. Satkhira lies in the southwest corner of Bangladesh, between the coordinates of 21°36′ and 22°54′ north latitude and 88°54′ and 89°20′ east longitude. It is linked to the north by Jessore and the south by the Gulf of Bengal (Fig. 1a). As shown in Fig. 1b, Satkhira is divided into seven upazilas (local administrative divisions): Assasuni, Shyamnagar, Kaliganj, Debhata, Satkhira Sadar, Tala, and Kalaroa. Satkhira has a total of 3817.29 square kilometres.

### Description of materials

**Figure 2:**
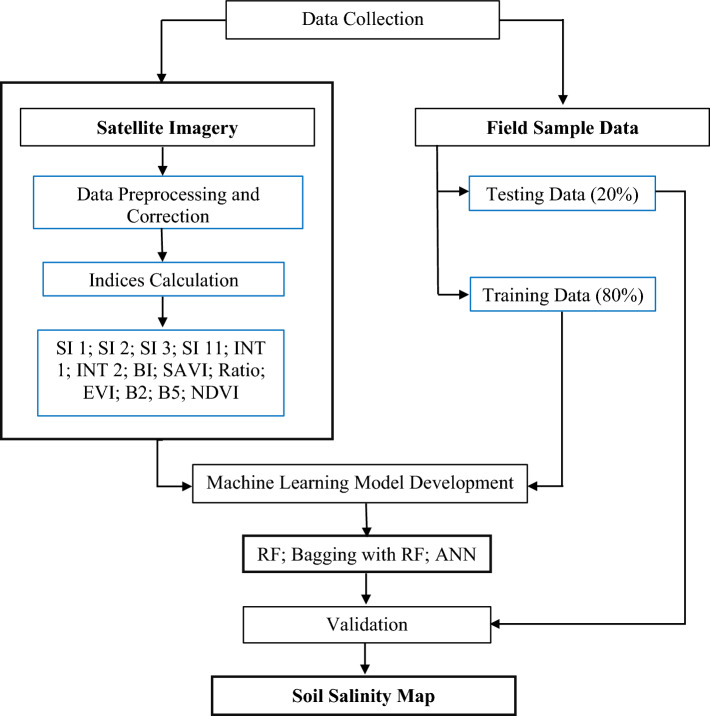
Methodological framework.

Thirteen remote sensing-based soil salinity indices were selected in this study based on a comprehensive literature review shown in Table 1. Indices were generated using the Landsat 8 OLI image (path 138, row 44, spatial resolution: 30 m) of 2021 and the equations used in^25, 26, 47^. Satellite images were pre-processed using geometry correction, atmospheric adjustment, and radiometric correction before the indices were calculated. Geometry correction is the process of aligning the satellite image to a reference map. This is important because it ensures that the image is correctly georeferenced, which is necessary for accurate measurements of spatial features. Geometry correction can be performed using a variety of methods, such as ground control points (GCPs) or a digital elevation model (DEM)^48^. The method of atmospheric adjustment involves taking atmospheric effects out of the satellite image. This is crucial because the spectral signature of objects in an image may be obscured or warped by atmospheric factors, which could result in miscalculated measurements. There are numerous techniques for atmospheric correction, including FLAASH and Dark Object Subtraction (DOS)^48^. In radiometric correction, the digital number (DN) values in the satellite picture are transformed into reflectance values. This is significant because radiometric correction guarantees accurate reflectance measurements since DN values are not inversely related to reflectance. A number of techniques, including calibration curves and empirical methods, can be used to accomplish radiometric correction^49^. These pre-processing techniques increased the precision of the soil salinity indicators that were calculated. The atmospheric adjustment eliminated atmospheric effects that could have obscured or warped the spectral signature of objects in the image, and the radiometric correction made sure that the reflectance values were precise. The geometry correction made sure that the indices were calculated over the proper areas. Spatial reference was used to accomplish the geometric corrections. In order to reduce ambient noise, FLAASH atmospheric correction was performed. After radiometric correction, digital number (DN) data were transformed to reflectance. 241 soil salinity sample data were collected from the^26^. All sample data was located in Kaliganj Upazilla of Satkhira District (Fig. 1).^26^ collected the sample data in shrimp and rice fields in the dry season of 2016 using a HI 8033 Portable Conductivity/TDS Meter. Of the sample data, 80% was used for training the model and 20% was reserved for testing the model's performance. Figure 2 shows the methodological framework of the study.

**Table 1:** comprehensive literature review for soil salinity indices selection.

Salinity indices	^50^	^51^	^47^	^52^	^53^	^37^	^25^
Salinity index one (SI 1)	✓	✓	✓			✓	✓
Salinity index two (SI 2)	✓	✓	✓	✓			✓
Salinity index three (SI 3)	✓	✓	✓	✓		✓	✓
Salinity index eleven (SI 11)	✓		✓				✓
Intensity index one (INT 1)	✓		✓				✓
Intensity index two (INT 2)	✓		✓				✓
Brightness index (BI)	✓	✓	✓	✓			✓
Soil adjusted vegetation index (SAVI)			✓		✓		✓
Ratio of two spectral bands		✓		✓			✓
Enhanced vegetation index (EVI)			✓				✓
Blue band (B2)		✓		✓		✓	✓
Near-Infrared band (B5)		✓					✓
Normalized difference vegetation index (NDVI)	✓		✓		✓		✓

The salinity index 1 (SI 1) calculates the variation in reflectance between the visible and near-infrared bands. It is determined by dividing (NIR−RED) by (NIR+RED). SI 1 can be used to map areas with high soil salinity because it is sensitive to salt^54^. Salinity Index 2 (SI 2) is comparable to SI 1, except instead of the red band, it utilizes the green band. As (NIR−GREEN)/(NIR+GREEN), it is calculated. High salinity areas can be mapped using SI 2, which is also sensitive to soil salinity^55^. The near-infrared to shortwave infrared reflectance ratio is used to calculate the SI 3 index. NIR/SWIR is used to calculate it. SI 3 can be used to map areas with high salinity since it is sensitive to the amount of water in the soil. The SI 11 index is an adjustment to the SI 3. It is determined by dividing (NIR−SWIR) by (NIR+SWIR). Greater precision can be achieved when mapping areas with high salinity using SI 11, which is more sensitive to soil salinity than SI 3^56^. The visible and near-infrared bands' intensity of reflectance is measured by the INT 1 index. The formula is (NIR+RED)2. INT 1 can be used to map areas of vegetative stress, which can be brought on by high salinity, even if it is not very sensitive to soil salt. Similar to INT 1, INT 2 substitutes the green band for the red band. The formula is (NIR+GREEN)2. INT 2 can be used to map areas of vegetative stress but is not very sensitive to soil salinity^27^. The BI index gauges how well the visible and near-infrared wavelengths reflect light. The formula is (3 × (NIR+Red+Green)). Although BI is not particularly sensitive to soil salinity, it can be used to map regions where vegetation is under stress. The normalized difference vegetation index (NDVI) has been modified by SAVI. It is determined using the formula (1 + L)*NDVI/(1 + L*L*NDVI), where L is a soil adjustment factor. SAVI can be used to map areas of high salinity with greater precision because it is more sensitive to soil salinity than NDVI^55^. Any index that is determined as the ratio of the reflectance of two spectral bands is referred to as the ratio of two spectral bands in general. The choice of the ratio to utilize will depend on the particular application. There are numerous alternative ratios that can be used. The NDVI was modified to create the EVI, which is intended to be less sensitive to soil brightness and more responsive to vegetation. (2.5 * NIR−1.5 * RED−0.5 * SWIR)/(2.5 * NIR + 1.5 * RED + 0.5 * SWIR) is the formula for calculating it. High salinity can result in areas of stressed vegetation, which can be mapped using EVI^57^. The blue band (B2) is a spectral band that gauges how well the Earth's surface reflects light in the visible blue spectrum. The Earth's surface's reflectance in the near-infrared region of the spectrum is measured by the near-infrared band (B5) of the spectrum. Finally, the difference between the reflectance of the near-infrared and red bands is measured by the commonly used index (NDVI). It is determined by dividing (NIR−RED) by (NIR+RED). NDVI can be used to map areas with vegetation because it is sensitive to vegetation^27^.

### Methods of soil salinity mapping

Three machine learning methods were applied in this study to predict soil salinity, and the description of these models is discussed in the following section.

#### Random forest

Breiman^58^ introduced RF, a popular ensemble learning strategy that may be used for tasks including classification, regression, clustering, and interaction detection. One decision tree is not a good classifier due to its high bias and variability. Nevertheless, because it may use ensemble trees to reduce these problems, RF typically results in more stable models. Many random binary trees are generated by RF to form the forest. A classification and regression tree (CART) is constructed by randomly selecting a set of variables at each node in a bootstrap sample. Each tree based on a bootstrap sample has an error rate known as the “out-of-bag” (OOB) error rate, which is calculated using data outside of the bootstrap sample. Class membership and model construction are ultimately decided by a vote of all trees (output). Adjusting the number of trees in the forest (ntree) and the number of variables assessed at each node (mtry) prior to running the RF model can help decrease out-of-the-box (OOB) error and boost model performance. To begin, RF generates a bootstrap sample of the training data. A bootstrap sample is a data sample formed by randomly sampling with replacement. This means that some data points may be chosen more than once, while others may be chosen just once. After generating a bootstrap sample, a decision tree is trained on it^58^. The greedy optimization technique is used to train the decision tree. Greedy optimization finds the best split at each node of the decision tree depending on data attributes. The process of developing a decision tree and training it on a bootstrap sample is repeated many times. The number of decision trees generated is an RF algorithm hyperparameter. The more decision trees generated, the more stable the RF model^59^. Bagging is a technique used in ensemble learning to reduce a model's variance. Bagging works by training multiple copies of a model using distinct bootstrap samples of training data. Individual model forecasts are then integrated to get a final projection^60^. Decision trees are supervised learning algorithms that can be used to perform classification or regression problems. Decision trees operate by iteratively grouping data into smaller and smaller groups until each group contains only data points from the same class. The decision rules used to partition the data are developed depending on the data's features^61^. Decision trees are supervised learning algorithms that can be used to perform classification or regression problems. Decision trees operate by iteratively grouping data into smaller and smaller groups until each group contains only data points from the same class. The decision rules used to partition the data are developed depending on the data’s features. A random binary tree is a sort of decision tree in which features are chosen at random at each node. The purpose of random binary trees is to reduce decision tree model variance by making it less sensitive to individual characteristics. Random forest first creates a bootstrap sample of the training data before generating random binary trees. A bootstrap sample is a data sample formed by randomly sampling with replacement. This means that some data points may be chosen more than once, while others may be chosen just once^58^.

#### Bagging with random forest

Bagging is a straightforward and very effective ensemble technique. The predictions of many machine learning algorithms may be combined using the ensemble technique, which has been shown to be more accurate than using any one model alone. In order to create a unique overall model, the Bagging method combines the Bootstrap and Aggregating algorithms^62^. The algorithm for labelling items is rather sensitive. Using the Bagging method, even slight modifications to the dataset will result in very different findings. Each learner's data is collected via bootstrap sampling, and then the estimated and combined ensemble is created using the learnt learner^63^. Bagging raises precision because it promotes more unsupervised learning. In this research, Bagging is combined with RF for predicting soil salinity mapping. Bagging is a bootstrap aggregation technique that can be used to improve the random forest model's effectiveness in forecasting soil salinity mapping. By training several decision trees on bootstrap samples of the training data, bagging reduces overfitting, increases variety, and improves the accuracy of the random forest model. Individual decision tree forecasts are then integrated to form a final prediction^64^.

#### Artificial neural network

ANN build their models of processes on top of previously observed behavioral patterns. It has several layers of organization, and processing units like neurons, as well as the three levels of input, covered, and output^65^. One layer are linked to those in the next by means of attachment weights. At the output of the middle layer, the data is sent on to the next layer (hidden layer). The input layer is responsible for taking in the data, while the output layer is responsible for producing the ultimate result of the ANN model. The input data is received by the intermediate layers, which then forward it to the appropriate nodes in the higher-level layers. Hidden layers take in a variety of inputs and use those weights to produce an intermediate output. The activation functions are used to calculate the outputs of the hidden and output layers in the ANN model. The output is determined by the sum of the input weights and the bias settings. Building the network and tweaking the link weights are the two meaty parts of an ANN modelling process. The research shows that water engineering is only one of several areas that uses the backpropagation training approach. The efficacy of an ANN model may be gauged by seeing how well it responds to input. After the model's weights have been established, the discrepancy between observed and predicted values can be reduced. As the output deviates from the observed value, the weights and biases are adjusted to reduce the error values. To compensate for the sluggish convergence rate of the backpropagation approach, this study used meta-heuristic optimization strategies. The fundamental unit of artificial neural networks is the biological neuron and its reduced qualities. These were created as a basic mathematical model that mimicked human brain activity^66^. It is made up of n inputs, yielding the vector x = (× 1,…. xn). The weight parameter, which can be positive or negative, is multiplied by each input. Another input neuron × 0 = 1 is rated by weight × 0, which indicates the bias. The sum of all weighted inputs yin reflects the neuron's intrinsic potential^67^:

1 $$Yin={\sum }_{i=0}^{n}WiXi$$

The neuron's potential is calculated using the following Eq. (1). The weighted sum is routed via a neuron activation function y = f (y in) to create the neuron's final output. This, in turn, can stimulate neurons in the neural network layer underneath. When neurons are joined together, they create a neural network. The linking mechanism is designed in such a manner that one neuron’s output becomes the input of another. The network’s neurons are grouped into layers^68^. Each network consists of an input layer, an output layer, and an unknown number of hidden layers. The capacity to change the weights of neurons is an important feature of neural networks. The network’s weights are reinforced or weakened based on correct or erroneous replies^67^. There are three types of learning algorithms: supervised, unsupervised, and reinforcement. A network model is a multilayer neural network that represents each neuron as a training method. In multilayer perceptrons, neuron activation functions are differentiable continuous functions, with the sigmoid function being the most commonly utilized^69^. The following equation no (1) is used calculate the potential of the neuron. The weighted sum is routed via a neuron activation function y = f (y in) to create the neuron's final output. This, in turn, can stimulate neurons in the neural network layer underneath. When neurons are joined together, they create a neural network. The linking mechanism is designed in such a manner that one neuron’s output becomes the input of another. The network's neurons are grouped into layers^68^. Each network consists of an input layer, an output layer, and an unknown number of hidden layers. The capacity to change the weights of neurons is an important feature of neural networks. The network’s weights are reinforced or weakened based on correct or erroneous replies^67^. Three categories of learning algorithms exist: supervised, unsupervised, and reinforcement learning. An example of a network model is a multilayer neural network where each neuron is a training method. Multilayer perceptron neuron activation functions are continuous, differentiable functions, with the sigmoid function being the most popular^69^.

2 $$f(x)=\frac{1}{1+{e}^{x}}$$

Equation (2) is used to calculate the sigmoid function. Multilayer perceptron result in complete neuron connectivity—each neuron in the layer is connected to all neurons in the preceding (following) layer^70^. Complex connections between input data and output predictions can be learned by ANNs: The complicated phenomena of soil salinity is regulated by a number of variables, such as climatic conditions, soil properties, and agricultural activities. In order to create precise predictions of soil salinity levels, ANNs can learn these intricate correlations from previous data. ANNs can deal with noisy or lacking data: Data on soil salinity are frequently erratic or lacking, as a result of things like sensor malfunctions and missing information. Even with imperfect data, ANNs can handle this sort of data effectively and still produce reliable predictions. ANNs may be scaled: Individual fields to whole areas can be predicted to have high soil salinity using ANNs. Complex connections between input data and output predictions can be learned by ANNs: The complicated phenomena of soil salinity is regulated by a number of variables, such as climatic conditions, soil properties, and agricultural activities. In order to create precise predictions of soil salinity levels, ANNs can learn these intricate correlations from previous data^71^. ANNs can deal with noisy or lacking data: Data on soil salinity are frequently erratic or lacking, as a result of things like sensor malfunctions and missing information. Even with imperfect data, ANNs can handle this sort of data effectively and still produce reliable predictions. ANNs may be scaled: Individual fields to whole areas can be predicted to have high soil salinity using ANNs. That is why ANN is very efficient for soil mapping and management^72^.

### Validation process

The ROC curve is a graphical illustration of sensitivity on the y-axis and specificity on the x-axis for various test data cut-off points. It is typically represented as a box with two axes, each of which runs from zero to one. The AUC is a sensitivity and specificity statistic that may be used to measure the intrinsic validity of a diagnostic test. If the diagnostic test has an AUC of 1, it can reliably distinguish between model soil salinity and field value. This means that there are no false positives or negatives, indicating that the sensitivity and specificity are both optimal. In actuality, this is highly unlikely to happen. The stronger the test performance, the closer the AUC is to one. The square is divided in half along the diagonal from (0, 0) to (1, 1), with each half being 0.5 square metres in size. The test has a 50/50 probability of successfully discriminating between soil salinity and non-soil salinity when the ROC is this line. Since AUC = 0 means that the test mistakenly identified all soil salinity participants as negative and all non-soil salinity individuals as positive, the minimal AUC value should be 0.5 rather than 0. When the test findings are reversed, area = 0 becomes area = 1, allowing an entirely erroneous test to be changed into a fully accurate test. The performance of models that predict soil salinity from sensor data assessed using the ROC curve and AUC in the context of soil salinity prediction. The percentage of soil salinity samples that are accurately identified as positive is the sensitivity. The percentage of non-soil salinity samples that are accurately categorized as negative is known as the specificity. The AUC is a measurement of how effectively the model can differentiate between samples with and without soil salinity. To calculate the sensitivity and specificity the following Eqs. (3) and (4) had been used^73^:

3 $${\text{Sensitivity}} = {\text{True}}\,{\text{Positives}}/\left( {{\text{True}}\,{\text{Positives}} + {\text{False}}\,{\text{Negatives}}} \right)$$

4 $${\text{Specificity}} = {\text{True}}\,{\text{Negatives}}/\left( {{\text{True}}\,{\text{Negatives}} + {\text{False}}\,{\text{Positives}}} \right)$$

Based on the required trade-off between sensitivity and specificity, the thresholds for distinguishing between soil salinity and non-soil salinity is selected. For instance, a threshold that has a high sensitivity but a low specificity in order to reduce the number of false negative is used. On the other hand, a threshold that has a low sensitivity but a high specificity in order to reduce the number of false positives is used.

## Results

### Descriptions of indices

**Figure 3:**
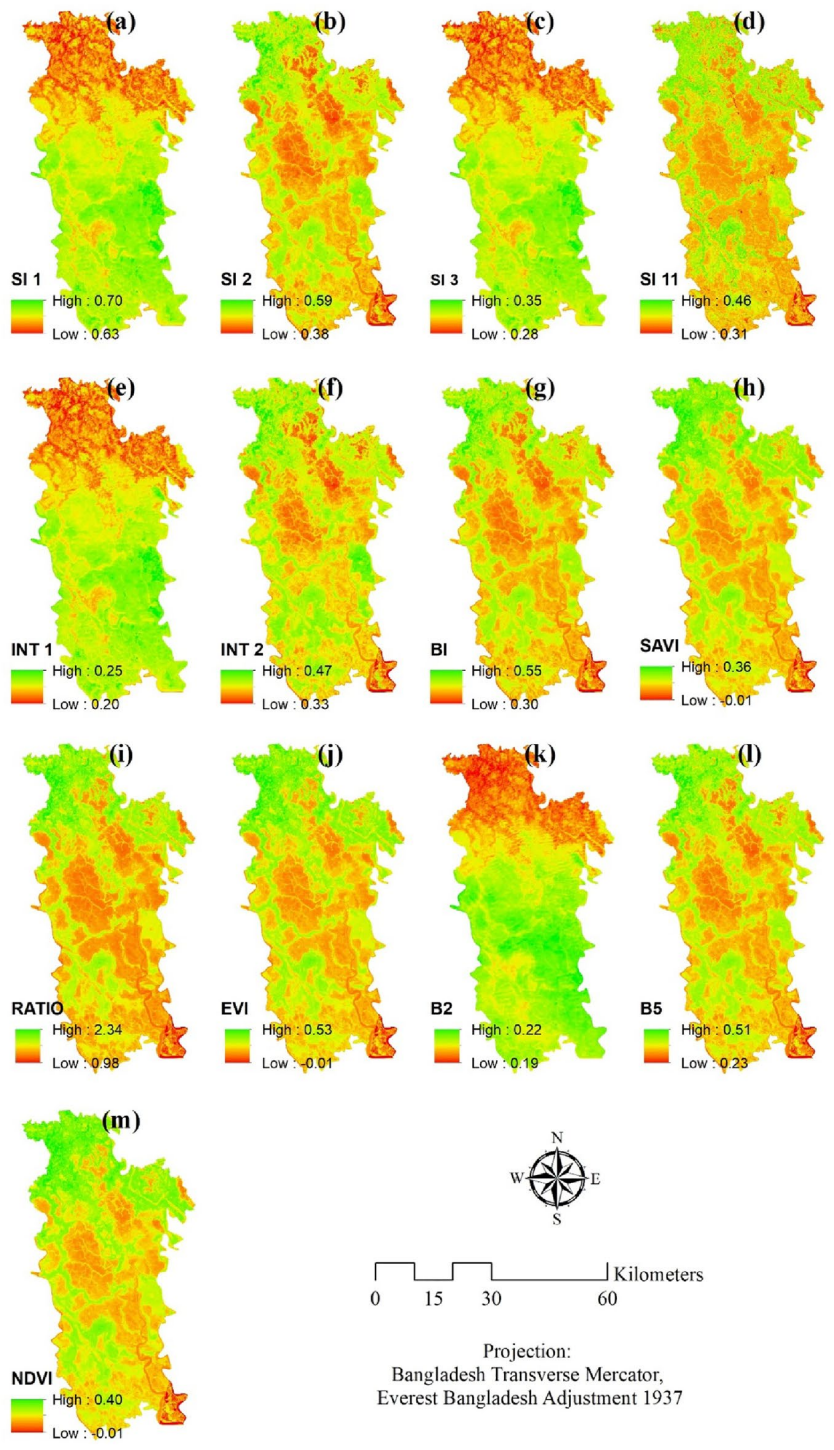
Spatial distribution of parameters, prepared by the authors using ArcGIS software version 10.5, (https://www.esri.com/en-us/arcgis/products); data was derived from Landsat 8 OLI image of USGS, (https://earthexplorer.usgs.gov/).

Among the selected 13 soil salinity indices 4 indices, namely SI 1, SI 3, INT 1 and B2, showed similar spatial distribution over the study space (Fig. 3), where relatively low values were found in the north as opposed to the central and southern part of Satkhira where moderate to high values were observed as a whole. However, the rest of the indices (SI 2, SI 11, INT 2, BI, SAVI, RATIO, EVI, B5 and NDVI) exhibited an unevenly scattered geographical distribution of values which were more or less similar in nature. The four indicators (SI 1, SI 3, INT 1, and B2) that displayed comparable geographical distributions are all affected by the soil’s salinity and moisture content. This is true because each of these indexes measures how much light the earth reflects^74^. The soil reflects more light when it is moist, and it also reflects more light when the soil has a high salt concentration. There is a known relationship between these indices and soil salinity. Studies have shown that SI 1, SI 3, INT 1, and B2 are all positively correlated with soil salinity. This means that as the value of these indices increases, the level of soil salinity also increases^75^. These indicators' geographical distribution shows that soil salinity is higher in the center and southern parts of Satkhira than in the north. This is most likely owing to the fact that the center and southern parts of Satkhira are located along the shore, making them more vulnerable to flooding and seawater intrusion.

The highest SI value was recorded as 0.70 for SI 1 (amongst SI 1, SI 2, SI 3 and SI 11) whereas the lowest was found for SI 3 (0.28). There was a mere difference between the two marginal values of INT 1 (0.20–0.25), indicating characteristics which are mostly alike and for INT 2 the highest and lowest values were estimated at 0.47 and 0.33 respectively. Again, BI, representing the brightness of the soil, was 0.55 calculated as the highest as compared to the lowest value of 0.30. The BI indices for most of the areas of Satkhira were found moderate to higher in values, suggesting a high association with the wetness and salt content of the soil.

As far as is observed, there was a significant difference between the highest and lowest values of NDVI, EVI and SAVI indices, which were mostly employed to measure the greenness of the geographical unit. The lowest value was − 0.01 for all of them as compared to the highest values of 0.40, 0.53 and 0.36 respectively. Since SAVI makes necessary corrections for soil brightness in areas with low vegetation cover and EVI reduces the canopy background noise with necessary atmospheric correction, all of these three indices (NDVI, EVI and SAVI) combined can determine the areas where the vegetation cover is dense and where it is not. A variety of variables, including local topography, geology, land use, climate, vegetation, soil type, and local characteristics or land use practices, might explain the varied regional distribution of soil salinity indices. All of these elements must be considered when creating reliable maps of soil salinity and designing solutions for reducing the consequences of soil salinity and safeguarding agricultural yield^75^.

### Output of models

**Figure 4:**
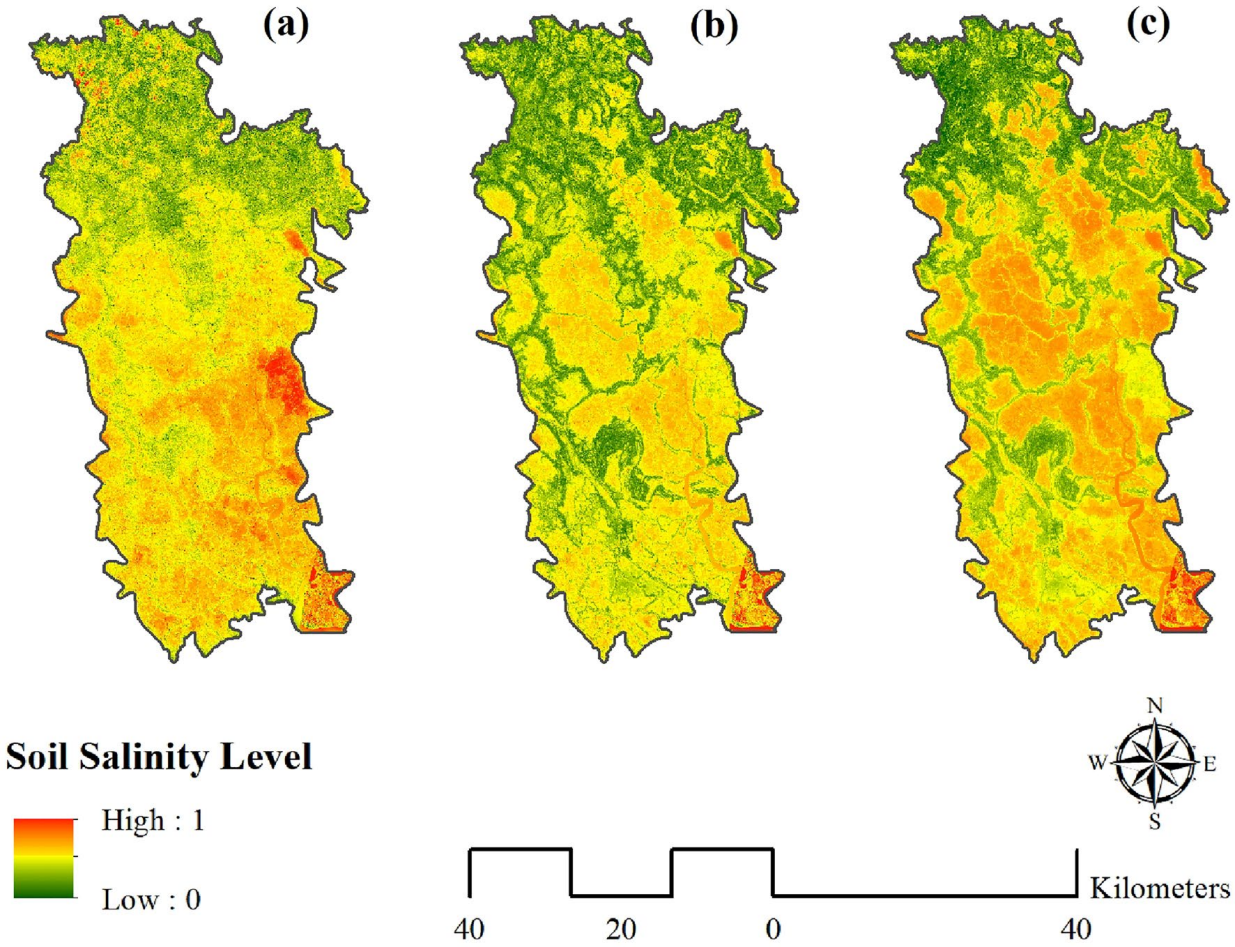
(**a**) RF; (**b**) Bagging with RF; (**c**) ANN, prepared by the authors using ArcGIS software version 10.5, (https://www.esri.com/en-us/arcgis/products).

Figure 3 shows three different maps for three distinct models viz. RF, bagging with RF, and ANN to highlight variations in soil salinity level of the study area with values ranging from 0 to 1 representing low to high soil salinity concentrations. The red zones indicate regions with high salinity, whereas the green zones indicate regions with low salinity. Between these two zones are certain zones of moderate salinity, indicated on the map by a combination of red and green colors In all three models Kalarola upazila contains a low salinity level compare to other upazila. The eastern part of the Tala upazila also contains a low salinity level (Fig. 4). From Fig. 3 it can be said that salinity indices like (SI 1, SI 3, INT 1, and B2) have low values in these regions which explains why the salinity level is comparatively here. On the other hand, Assasuna and Shyamnagar have comparatively higher salinity level. It happened due to the high indicator value of (SI 1, SI 3, INT 1, and B2) in this region. So, it can be said that these indicators have positive correlation with the soil salinity. As the value of the indicators increase so do the value of the soil salinity. Negative correlation can be found in the indicators like (BI, SAVI, Ratio, EVI, and INT2). There are differences in the results of three models for example: Debhata and parts of Satkhira sadar have higher salinity level in ANN compare to rest of the model. Moreover, river and its surroundings shows comparatively higher salinity level in ANN. It can be said that ANN shows a better correlation with indicators than rest of the model as indices like (SI 1, SI 3, INT 1, and B2) shows high value in these regions and (BI, SAVI, Ratio, EVI, and INT2) shows low value in these regions. From the output of these three models it can be perceived as a whole that the northern side of Satkhira district is exposed to low soil salinity concentration as opposed to the central and southern part of Satkhira where moderate to high soil salinity are observed.

### Validation of models

**Figure 5:**
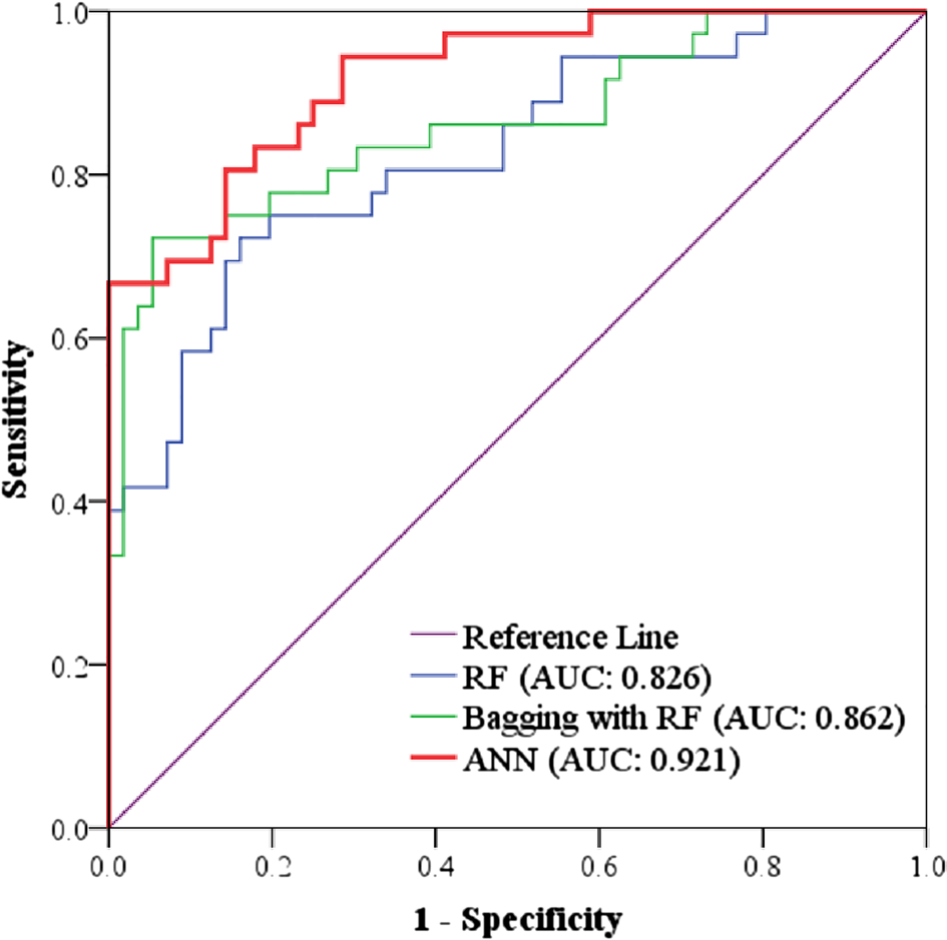
Validation of soil salinity models.

The true positive rate (TPR) vs the false positive rate (FPR) at various threshold values is plotted graphically as the (ROC) curve. The area under the ROC curve, or AUC, is a measurement of a binary classifier's overall performance. A perfect classifier has an AUC of 1.0, whereas a random classifier has an AUC of 0.5. ANN model's high AUC value indicates two ways in which it is successful in forecasting soil salinity zones. In the following table RF, Bagging with RF and ANN models have been identified as test result variables with their relative test statistics. Here area under curve (AUC) at 95% confidence interval (CI) for each model are calculated. Compared to other two models the ANN model significantly shows superior promise to predict and identify soil salinity zones as it has the maximum value (0.921) of AUC (Fig. 5). First, compared to the RF and Bagging with RF models, the ANN model was able to properly identify a larger percentage of soil salinity zones. Second, compared to the RF and Bagging with RF models, the ANN model was able to attain a reduced false positive rate. For the purpose of validation these models have been checked by ROC curve. As a result, the ANN model was less likely to mistakenly label a soil sample as salinous when it wasn’t. For stakeholders and policymakers, this confirmation has important practical ramifications. The ability of the ANN model to accurately forecast soil salinity zones may be utilized to guide decisions on the use of land and the management of water resources. The ANN model, for instance, might be used to pinpoint sections of land that are vulnerable to soil salinity and to create plans to counteract its impacts.

### Soil salinity zones

**Figure 6:**
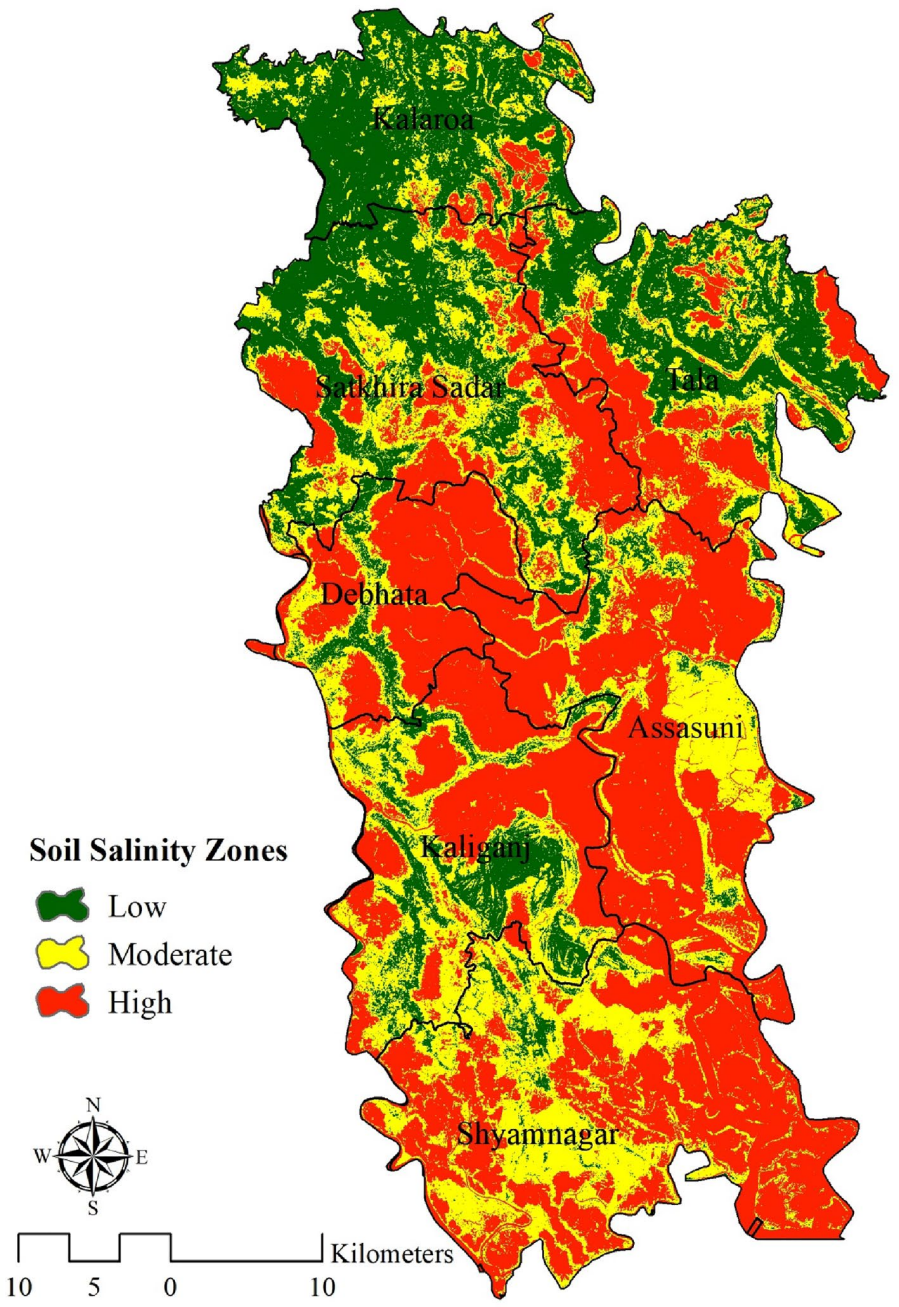
Spatial distribution of soil salinity zoning, prepared by the authors using ArcGIS software version 10.5, (https://www.esri.com/en-us/arcgis/products).

The output obtained from the ANN model is selected in this study to define the soil salinity zones of Satkhira district since it has the highest prediction accuracy (0.921). Moreover, the indicators such as (SI 1, SI 3, INT 1, and B2) are positively correlated with the salinity level map (Fig. 3) where salinity level is high and (BI, SAVI, Ratio, EVI, and INT2) are highly correlated with the areas where salinity level is low in the ANN model (Figs. 3 and 4). So it can be said that the high correlation is another important factor to choose ANN as the preferred model. Additionally, robustness and interpretability of the model are also taken in consideration while choosing this model. The whole study area is divided into three zones i.e. low, moderate, and high as per their soil salinity concentrations (Fig. 6). The areal extent of high soil salinity is recorded as 977.94 km^2^ which is about 43.51% of the total study region (Table 2). Additionally, 30.56% area of Satkhira is exposed to moderate soil salinity (686.92 km^2^), followed by 25.93% area share of low soil salinity (582.73 km^2^) concentrations. As discussed earlier the level of soil salinity in the northern area of Satkhira district is comparatively lower than that in the central and southern regions. Moderate soil salinity zones are scattered almost all over the study space in an irregular manner, although greater concentration of moderate soil salinity is observed in the south western part of Satkhira district. Lastly, central Satkhira and south eastern side of Satkhira are experiencing high level of salinity concentration as it is observed from the model output. Most of the part of Shayamnagar, Debhata and Assasuni contains high salinity zones. While some parts of Shayamnagar and Assasuni contains moderate salinity zone. On the contrary, low salinity zone can be found in the Tala, Satkhira sadar and Kalarola upazila. Numerous agricultural planning, resource management, and policy initiatives can benefit from the identification of soil salinity zones in the Satkhira district. For instance, the data can be used to pinpoint areas where salt-tolerant plants can thrive, create water management strategies that use less salty water, give farmers financial aid to help them adopt salt-tolerant plants or put in water treatment systems, and create regulations that encourage the use of salt-tolerant plants and water-saving techniques. Stakeholders may reduce the detrimental effects of soil salinity on agricultural productivity in Satkhira area by implementing these actions.

**Table 2:** Distribution of soil salinity zones.

Soil salinity	Area in km^2^	Area in percent
Low	582.73	25.93
Moderate	686.92	30.56
High	977.94	43.51
	2247.58	100.00

## Discussion

This study compared the performance of three different models, namely RF, Bagging with RF, and ANN, in terms of their ability to predict the level of soil salinity prevalent in the study area, which was the Satkhira district of Bangladesh. For this purpose, 13 soil salinity indices were calculated based on the Landsat 8 OLI image and 241 soil salinity sample data were collected from the^26^ (80% used as training data and 20% as test data). These data were then tested through the 13 indices with the help of the three machine learning models mentioned above. Firstly, 13 indices map were created using Arc GIS software. Raster calculator had been used to calculate the value of each indicator then the maps were created (Fig. 2).

According to the results, indicators like (SI 1, SI 3, INT 1, and B2) showed high values in Shayamnagar, Debhata and Assasuni whereas the low values have been observed in the upazilas like Tala, Satkhira sadar and Kalarola upazila. On the contrary, (SI 1, SI 3, INT 1, and B2) have showed the opposite type of result. The rest of the indicators result were moderate all over the region.^27^ have used the same type of indices and the result was quite similar (Fig. 3). After that salinity level distribution was done using three machine learning algorithms namely RF, Bagging with RF, and ANN. 80% training data has been used to create the three different map. Previous studies related to machine learning models have used the same type of procedure^70^. Kalarola upazila has minimal salinity in all three models. The eastern side of Tala upazila has minimal salinity (Fig. 4). Figure 3 shows that salinity indices such SI 1, SI 3, INT 1, and B2 are low in these places, explaining the low salinity level. On the other side, Assasuna and Shyamnagar are saltier due to high indicator values (SI 1, SI 3, INT 1, and B2) in this region. Thus, these indicators positively correlate with soil salinity. As indicators rise, soil salinity rises. BI, SAVI, Ratio, EVI, and INT2 have negative connection. In some aspects models have showed different results: Debhata and sections of Satkhira sadar have higher ANN salinity than the rest of the model. The river and its environs are also saltier in ANN. ANN has a greater correlation with indicators than the remainder of the model since (SI 1, SI 3, INT 1, and B2) have high values in these regions while (BI, SAVI, Ratio, EVI, and INT2) have low values^25^ After that the rest of 20% of the sample points had been used to predict the models. Results showed that RF had a ROC value of 82%, RF with bagging had a success rate of 86% and 92% of the success rate was showed by ANN model (Fig. 5). All the model results were accurate but as the accuracy of the ANN was bit higher than other model so ANN was used. Moreover indicators were highly correlated with the salinity level map of ANN (Figs. 3 and 4). Artificial neural networks (ANNs) can learn complex nonlinear correlations between soil salinity and other environmental parameters, making them stronger salinity mapping models. This matters because soil salinity is complicated and affected by climate, geology, and land use^76^. Previous studies done by^33, 64, 76^ have preferred the same model over other machine learning models. According to the ANN model output almost 74.07% area of Satkhira district was exposed to moderate to high soil salinity concentrations. Similar type of result can be found in the study done by^26, 37^. High salinity percentage in the study area was around 43% which is almost half of the total study area (Fig. 6). High salinity can be found in Shayamnagar, Debhata and Assasuni upazila^27^. In the dry season, P1 pH ranged from 6.5 to 7.5, whereas P2 pH ranged from 6.2 to neutral (7.2) in these areas which is quite alarming^77^. Morshed et al.^26^ have recommended to do only cultivate shrimp as the upazilas contain high level of salinity and not appropriate for other crops. Moderate salinity zone is around 30.56% and it can be found in parts of Shayamnagar, Kaliganj and Assasuni. Sarkar et al.^27^ have found similar kind of findings and Morshed et al.^25^ have proposed mixed agriculture such as rice with shrimp as the salinity is moderate here. But using partial least regression model^27^ have found that the eastern part of Assasuna contains very low salinity zone but in this study it is defined as moderate saline zone. It can happen as^27^ have classified the study area in five different zone but in this study it is classified into 3 different zones. Low potential zone was around 25.93% and it was found in the upazilas like Tala, Satkhira sadar and Kalarola. Previous study done by^27^ have identified parts of Satkhira sadar and Kalarola as low saline zone but don’t Tala as a low saline zone. But study done by^25, 26, 78^ have identified all three upazilas as low saline zone. Moreover^25, 78^ have recommended these regions for different type of crop cultivation as the salinity level is very low. Pervious researchers using ANN^36, 37, 76^ for soil salinity have also found that areas close to ocean, soil made up with clay and silt, are both good at absorbing salt. It is also found that areas like Satkhira where temperature is hot and humid can contribute to the evaporation of water and the concentration of salt in the soil^79^.

Soil salinity zones can assist stakeholders in making sustainable land and water management decisions^80^. It can be used to identify locations appropriate for different types of agricultural production, manage water resources, and guide soil salinity policy actions^81, 82^. Land use and crop selection are also affected by soil salinity zones,^26, 78^ have already made land zoning for different crops using a salinity zoning map. However, past studies have not employed machine learning algorithms to evaluate soil salinity zones. This study is the inaugural investigation into evaluating salinity levels through utilizing three distinct machine learning models. This research can provide valuable insights for farmers, policymakers, and environmental specialists, enabling them to gain greater confidence in the outcomes.

Additionally, it will aid in their comprehension of which model is most effective in detecting soil salinity. Furthermore, this study may catalyze other researchers to conduct similar analyses. However, this study has some limitations. Ground truth points play a vital role in training and testing data and differs through region. So, ANN can show the best result due to the geographic location and factors of Satkhira, which might be applicable for other areas. Sometimes, Machine learning models require a large amount of accurate and representative data to train. They can also overfit the training data and be difficult to interpret. This study addresses these limitations by data preprocessing, model selection, model tuning, ensemble learning, and carefully selecting ground truth points.

## Conclusion

The present work employs machine learning techniques and remote sensing technology to cartographically represent soil salinity levels throughout the Satkhira district of Bangladesh. The evaluation encompassed three distinct machine learning models: random forest (RF), bagging with RF, and artificial neural network (ANN). The artificial neural network (ANN) model demonstrated the highest level of accuracy, achieving a success rate of 92%. The findings indicate that a significant proportion, around 74%, of the Satkhira district has moderate to high soil saline concentrations. Shayamnagar, Debhata, and Assasuni upazilas were identified as locations with high saline levels of over 40%. Parts of Shayamnagar, Kaliganj, and Assasuni upazilas were observed to exhibit moderate salt levels ranging from 20 to 40%. Tala, Satkhira sadar, and Kalarola upazilas showed low salt levels below 20%.

Utilizing machine learning and remote sensing techniques in soil salinity mapping offers numerous benefits. Initially, employing this method proves to be a financially viable and highly successful approach for comprehensively assessing soil salinity across extensive regions. Additionally, remote sensing can be utilized to delineate soil salinity levels in areas that provide challenges in terms of accessibility or where conventional soil sample methods are impracticable. Moreover, this technology has the potential to provide real-time mapping of soil salinity, thereby enabling the monitoring of soil salinity variations resulting from climate change or other influencing variables. The study’s findings’ ramifications are significant regarding sustainable land and water management in the Satkhira district. The results can be utilized to ascertain regions conducive to various agricultural practices, effectively administer water resources, and inform policy measures about soil salinity. The study’s approach and findings can be extrapolated and applied to other coastal regions with comparable soil salinity issues. Future research could address the shortcomings of this study. Initially, the analysis was carried out within a singular district in Bangladesh.

Further investigation is warranted in additional coastal areas to ascertain the results' generalizability to diverse contexts. Furthermore, the study solely considered a restricted range of variables that may influence soil salinity. Subsequent investigations ought to encompass a broader spectrum of variables, including but not limited to climate change, land utilization, and irrigation methodologies. Furthermore, the study employed a limited number of three machine learning models. It is recommended that future investigations undertake an evaluation of alternative machine learning models to assess their efficacy in the context of soil salinity mapping. Despite these constraints, this investigation's results offer significant perspectives on the mapping of soil salinity in the Satkhira district and similar coastal areas. The study's methodology and findings could enhance the precision and practicality of soil salinity predictions in the regions mentioned earlier.

## Data Availability

The datasets used in the study are available from the corresponding author upon reasonable request.
